# Fibrin-Hyaluronic Acid Hydrogel (RegenoGel) with Fibroblast Growth Factor-18 for In Vitro 3D Culture of Human and Bovine Nucleus Pulposus Cells

**DOI:** 10.3390/ijms20205036

**Published:** 2019-10-11

**Authors:** Sonja Häckel, Mona Zolfaghar, Jie Du, Sven Hoppe, Lorin M. Benneker, Nathalie Garstka, Marianna Peroglio, Mauro Alini, Sibylle Grad, Avner Yayon, Zhen Li

**Affiliations:** 1 AO Research Institute Davos, 7270 Davos, Switzerland; sonja.haeckel@insel.ch (S.H.); mona_zolfaghar@yahoo.com (M.Z.); du.jie@aofoundation.org (J.D.); marianna.peroglio@aofoundation.org (M.P.); mauro.alini@aofoundation.org (M.A.); sibylle.grad@aofoundation.org (S.G.); 2 Department of Orthopaedic, Inselspital Bern, 3010 Bern, Switzerland; Sven.Hoppe@insel.ch (S.H.); lorin.benneker@insel.ch (L.M.B.); 3 Universitätsklinik Wien, 1090 Vienna, Austria; nathalie.garstka@meduniwien.ac.at; 4 ProCore Ltd. Weizmann Science Park, 76400 Ness Ziona, Israel

**Keywords:** fibrin-hyaluronic acid hydrogel, fibroblast growth factor 18, nucleus pulposus cells

## Abstract

We investigated the effects of a fibrin-hyaluronic acid hydrogel (FBG–HA) and fibroblast growth factor 18 (FGF-18) for nucleus pulposus (NP) regeneration. Healthy bovine (*n* = 4) and human degenerated NP cells (*n* = 4) were cultured for 14 days in FBG-HA hydrogel with FGF-18 (∆51-mutant or wild-type) in the culture medium. Gene expression, DNA content, and glycosaminoglycan (GAG) synthesis were evaluated on day 7 and 14. Additionally, histology was performed. Human NP cells cultured in FBG-HA hydrogel showed an increase in collagen type II (COL2) and carbonic anhydrase XII (CA12) gene expression after 14 or 7 days of culture, respectively. GAG release into the conditioned medium increased over 14 days. Healthy bovine NP cells showed increased gene expression of ACAN from day 7 to day 14. Wild type FGF-18 up-regulated CA12 gene expression of human NP cells. Histology revealed an increase of proteoglycan deposition upon FGF-18 stimulation in bovine but not in human NP cells. The FBG-HA hydrogel had a positive modulatory effect on human degenerated NP cells. Under the tested conditions, no significant effect of FGF-18 was observed on cell proliferation or GAG synthesis in human NP cells.

## 1. Introduction

During the early degeneration process of the intervertebral disc (IVD), the nucleus pulposus (NP) as the inner, gelatinous part of the IVD loses the notochordal cells, which are replaced by more mature chondrocyte-like NP cells [1]. This mainly leads to a change in the extracellular matrix (ECM), whereby water-binding proteoglycans (PG) and collagens such as collagen type II are replaced by a more fibrous, stiff matrix mainly consisting of collagen type I [2]. This naturally degenerative process is not painful per se, as there are many patients with radiographic signs of IVD degeneration who do not suffer from back pain [3]. Nevertheless, there is a connection between disc degeneration and low back pain—the degenerative process causes an inflammatory environment in the IVD [4] and nerve ingrowth into the NP [5]. This leads to the clinical term degenerative disc disease (DDD). The “painful disc” or “discogenic pain” is described to be the main cause of low back pain in 26–42% of cases [6,7]. Besides surgical treatment, there is neither a minimally invasive treatment of discogenic pain, nor a possibility of drug-induced regeneration of the disc. Many growth factors have been investigated in pre-clinical studies [8,9,10]. Despite their promising in vitro results, there are still no clinical uses of growth factors for disc regeneration [10].

Fibroblast growth factor (FGF)-18 was first described in 1998 [11]. It acts as a signal protein in many physiological processes including chondrogenesis and skeletal development [12], where it stimulates anabolic processes in chondrocytes [13]. In the past years, a recombinant form of FGF-18 (rhFGF-18, Sprifermin) has been used as an experimental drug for the treatment of osteoarthritis (OA) in the knee joint [14,15,16,17]. This approach was based on the strong chondrogenic effect of FGF18 on human articular chondrocytes in vitro. Aside from this, a missense mutation leading to a constantly active FGF receptor 3 (FGFR3), the authentic receptor for FGF18, was discovered to be the cause of the genetic short limb dwarfism, achondroplasia (ACH) [18]. Because of the low incidence of OA in patients with ACH, it was suggested that the ACH mutation may protect against OA [19].

The signaling of all members of the FGF family occurs through the FGF receptors (FGFR). FGFR are transmembrane receptors, which can be classified into four types—FGFR1–4 [20]. They contain an extracellular ligand-binding domain, a transmembrane domain, and a cytoplasmic domain containing a split tyrosine kinase (sub)domain [21]. The FGF-18 wild type (WT) binds to its receptors with different affinities—FGFR3c > 4∆ > 2c > 1c >> 3b [20]. As the binding of FGF-18 to the FGFR3 leads to its chondrogenesis signaling-cascade [22], we previously developed an FGF-18 mutant (FGF-18 ∆51) to increase the selectivity of FGF-18 to FGFR3 [23]. To our knowledge, the potential regenerative effect of FGF-18 on NP cells is still unknown [10].

For an intradiscal growth factor delivery, a hyaluronic acid (HA)-based hydrogel has been suggested to be an appropriate carrier material. HA is a natural component of the NP ECM, and HA-based hydrogels with high molecular weight have shown to decrease inflammation in both NP [24,25,26] and cartilage tissue [27]. Fibrinogen–HA conjugate (FBG-HA), is water-soluble and polymerizes by cross-linking upon the addition of thrombin. This injectable viscoelastic hydrogel (RegenoGel) has already been approved for clinical use by the Israel Ministry of Health for intra-articular application in osteoarthritic knee joints [28].

Many similarities exist between the NP tissue and articular cartilage, including the hypoxic environment [29], the ECM composition [30], low cellularity [31], and a similar cell phenotype [32], suggesting possible similar effects of regenerative factors on chondrocytes and NP cells. Here, we studied the potential of both FGF-18 (wild type and ∆51 mutant) and the FBG-HA hydrogel for NP regeneration by analyzing gene expression of NP matrix components and NP cell phenotypic markers, as well as the effect on ECM production in healthy bovine and mildly degenerated human NP-derived cells.

## 2. Results

### 2.1. Expression of the FGF Receptors 1−4 in Bovine and Human NP Cells

**Figure 1:**
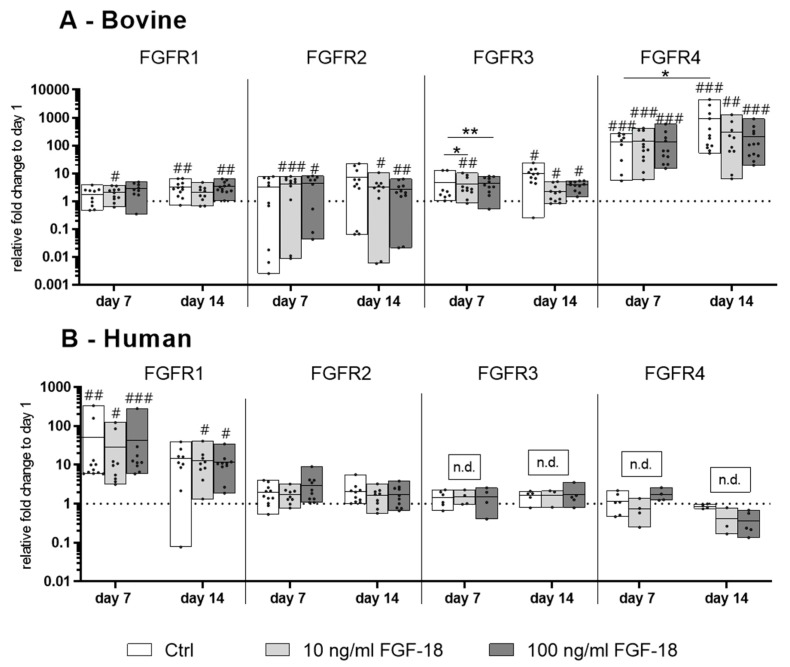
Relative mRNA expression of fibroblast growth factor (FGF) receptor 1−4 of (**A**) bovine (healthy) nucleus pulposus cells and (**B**) human (mildly degenerated) nucleus pulposus cells encapsulated in fibrinogen– hyaluronic acid conjugate (FBG-HA) hydrogel and stimulated with 10 or 100 ng/mL FGF-18 (FGF-18 ∆51 for bovine and FGF-18 wild type (WT) for human nucleus pulposus (NP) cells), after 7 or 14 days of culture. Data were normalized to the expression level of cells at day 1 in hydrogel culture. *n* = 4 (in human NP cells, two out of four donors did not show expression of FGFR3 and FGFR4 at the mRNA level, indicated as n.d. = not detected), # *p* < 0.05, ## *p* < 0.01, ### *p* < 0.001 vs. day 1, * *p* < 0.05, ** *p* < 0.01.

In healthy bovine NP cells, all FGF receptors (FGFR1–4) were expressed on the mRNA level. Encapsulation in hydrogel showed an upregulation of the FGFR1, -3, and -4 after 14 days of culture compared to the expression level on day 1. The FGFR4 showed the highest increase of gene expression over time (*p* < 0.001 vs. day 1). Additional stimulation with FGF18 ∆51 mutant showed a significant down-regulation of the FGFR3 after 7 days of culture (*p* < 0.05 for 10 ng/mL; *p* < 0.01 for 100 ng/mL) compared to the control group on day 7. No significant effects on FGFR1, -2, and -4 gene expression was detected with 10 and 100 ng/mL FGF18 ∆51 compared to the control (Figure 1A).

Human mildly degenerated NP cells showed a different FGFR expression profile compared to bovine cells. FGFR1 was significantly upregulated by encapsulating cells in hydrogel over 7 days (*p* < 0.01 vs. day 1). FGFR3 and -4 were detected on day 1 in all the four donors. After 7 or 14 days of culture, two donors of Pfirrmann grade 2 and 3 did not express FGFR3 and FGFR4, whereas another two donors at Pfirrmann grade 2 maintained comparable gene expression levels of FGFR3 and FGFR4 compared with day 1. These results indicate that human mildly degenerated NP cells can lose the FGF receptor expression after in vitro three dimensional (3D) culture within FBG-HA hydrogel. FGF-18 WT stimulation did not show any effect on the gene expression level of FGFRs 1−4 compared to the unstimulated control (Figure 1B).

### 2.2. Expression of NP Cell Markers in Bovine and Human NP Cells

**Figure 2:**
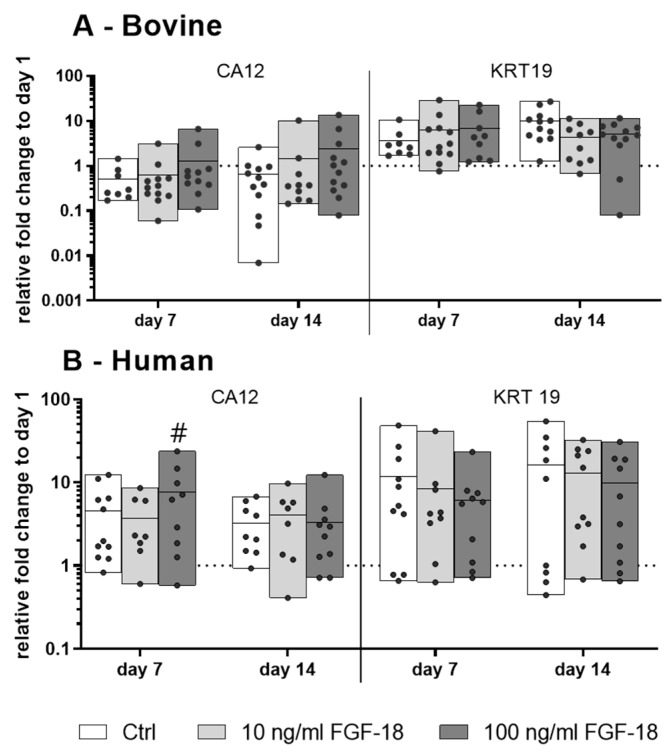
Relative mRNA expression of NP cell marker genes carbonic anhydrase 12 (CA12) and keratin 19 (KRT19) of (**A**) bovine (healthy) nucleus pulposus cells and (**B**) human (mildly degenerated) nucleus pulposus cells encapsulated in FBG-HA hydrogel and stimulated with 10 or 100 ng/mL FGF-18 (FGF-18 ∆51 for bovine and FGF-18 WT for human NP cells), after 7 or 14 days of culture. Data were normalized to the expression level of cells at day 1 in hydrogel culture. *n* = 4, # *p* < 0.05 vs. day 1.

The gene expression of the NP cell markers carbonic anhydrase 12 (CA12) and keratin 19 (KRT19) in healthy bovine NP cells did not change significantly over 14 days of culture with or without FGF-18 ∆51 stimulation (Figure 2A). On the other hand, mildly degenerated human NP cells showed a significant increase of CA12 gene expression upon stimulation with 100 ng/mL FGF-18 WT on day 7, which indicated a positive modulation of the NP cell phenotype (Figure 2B). KRT19 expression showed a trend of increase in bovine and human NP cells cultured in FBG-HA hydrogels after 7 and 14 days, although this was not significant.

### 2.3. Gene Expression of IVD Matrix Molecule and Catabolic Markers in Bovine and Human NP Cells

**Figure 3:**
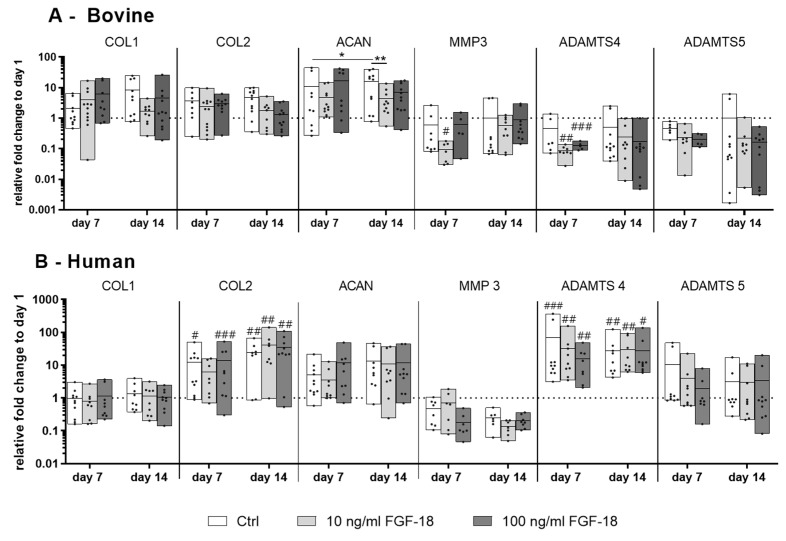
Relative mRNA expression of collagen type I (COL1), collagen type II (COL2), aggrecan (ACAN), and the catabolic markers matrix metalloproteinase-3 (MMP3) and a disintegrin and metalloproteinase with thrombospondin motif 4 and 5 (ADAMTS4 and ADAMTS5) of (**A**) bovine (healthy) nucleus pulposus cells and (**B**) human (mildly degenerated) nucleus pulposus cells encapsulated in FBG-HA hydrogel and stimulated with 10 or 100 ng/mL FGF-18, after 7 or 14 days of culture. Data were normalized to the expression level of cells at day 1 in hydrogel culture. *n* = 4, # *p* < 0.05, ## *p* < 0.01, ### *p* < 0.001 vs. day 1, * *p* < 0.05, ** *p* < 0.01.

There was no significant change in gene expression of collagen type I (COL1) and collagen type II (COL2) in healthy bovine cells with or without FGF-18 ∆51. After 14 days of culture, there was a significant increase in the aggrecan (ACAN) gene expression in FBG-HA hydrogel culture (*p* < 0.05 day 7 vs. day 14). By adding 10 ng/mL FGF-18 ∆51, this up-regulation effect was significantly diminished (*p* < 0.01), but it was still higher than the level on day 1. The catabolic gene marker matrix metalloproteinase-3 (MMP3) was significantly downregulated (compared to day 1) by adding 10 ng/mL FGF-18 ∆51 (*p* < 0.05) after 7 days. The catabolic marker a disintegrin and metalloproteinase with thrombospondin motif 4 (ADAMTS4) was significantly downregulated (compared to day 1) after 7 days with both tested concentrations of FGF-18 ∆51 (*p* < 0.01 at 10 ng/mL, and *p* < 0.001 at 100 ng/mL). ADAMTS5 showed no significant changes in gene expression over 14 days. Aside from this, no significant changes in ADAMTS5 were seen between the control and the FGF-18 groups (Figure 3A).

In human NP cells, the COL1 gene expression did not significantly change over time with or without FGF-18 WT (Figure 3B), whereas COL2 was significantly upregulated within all groups except for 10 ng/mL FGF-18 WT compared to day 1. ACAN expression level showed a donor-dependent increase after 14 days of culture in FBG-HA hydrogel, although it was not significant (Figure 3B). FGF-18 WT did not show a significant effect on the expression of COL2 or ACAN in human NP cells. The catabolic marker MMP3 was insignificantly downregulated by adding FGF-18 WT. Compared with healthy bovine NP cells, ADAMTS4 showed a contrary expression pattern. All tested groups showed a significant upregulation of ADAMTS4 after 7 and 14 days. No significant differences of ADAMTS5 gene expression could be detected after 7 and 14 days of culture (Figure 3B).

### 2.4. Proliferation and Matrix Protein Synthesis of Bovine and Human NP Cells

**Figure 4:**
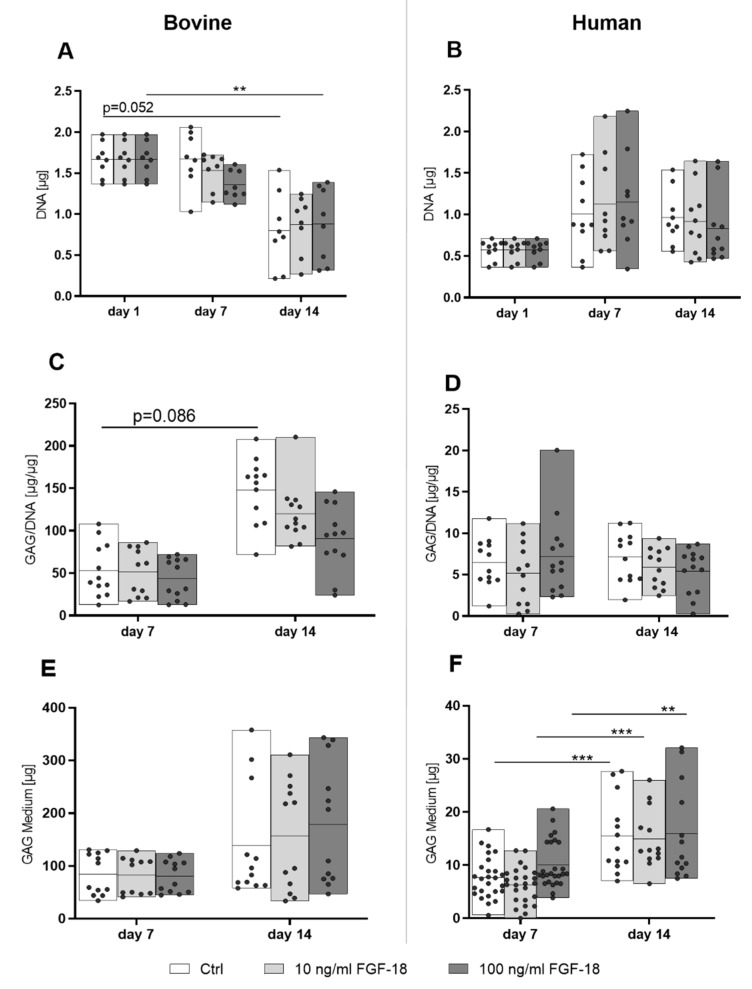
DNA (**A**,**B**), glycosaminoglycan (GAG)/DNA in hydrogel (**C**,**D**), and GAG content in medium (**E**,**F**) for healthy bovine nucleus pulposus cells (**A**,**C**,**E**) and mildly degenerated human nucleus pulposus cells (**B**,**D**,**F**) encapsulated in FBG-HA hydrogel and stimulated with 10 or 100 ng/mL FGF-18 after 7 or 14 days of culture. *n* = 4, ** *p* < 0.01, *** *p* < 0.001.

DNA content was determined as an indicator of cell proliferation. Over 14 days, bovine NP cells showed a decrease in measured DNA content, which was not influenced by FGF-18 (Figure 4A). Conversely, the DNA content in human NP cells remained constant, and the addition of FGF-18 did not induce significant changes in DNA content (Figure 4B).

The glycosaminoglycan (GAG) content in each hydrogel with encapsulated cells was normalized to its DNA content (GAG/DNA, Figure 4C,D). The GAG release into the conditioned medium is shown in Figure 4E,F. The GAG production from bovine healthy NP cells was approximately 10-fold higher compared with the human mildly degenerated NP cells during the 14-day culture period. In bovine NP cells, the GAG/DNA value in the control group (unstimulated) showed a trend to increase over time (Figure 4C), whereas the GAG content in medium (GAG accumulation over 7 and 14 days) showed no significant changes after 14 days, with or without FGF-18 stimulation (Figure 4E). In mildly degenerated human NP cells, there were no significant differences in GAG/DNA values in hydrogels (Figure 4D). The GAG content in the culture medium significantly increased over time, but did not differ between groups with or without FGF-18 WT (Figure 4F; 0 and 10 ng/mL *p* < 0.001; 100 ng/mL *p* < 0.01).

### 2.5. Histology Staining

**Figure 5:**
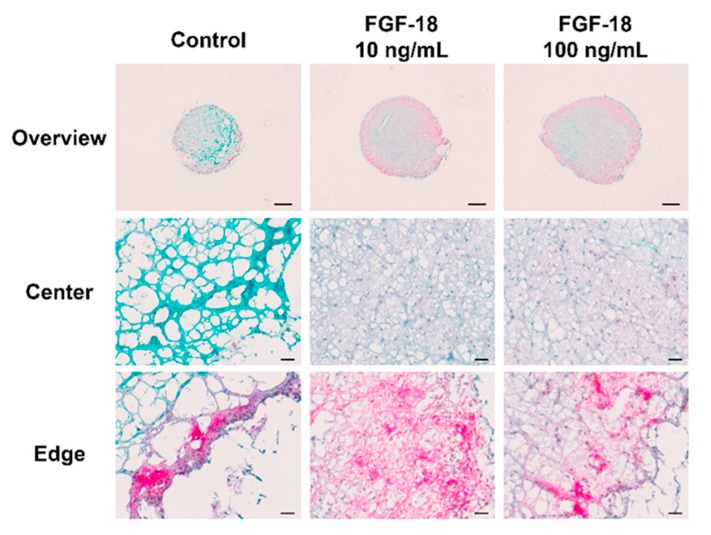
Representative Safranin O/Fast Green-stained transverse sections of bovine (healthy) nucleus pulposus cells encapsulated in FBG-HA hydrogel and stimulated with 0, 10, or 100 ng/mL FGF-18 after 14 days of culture. The red and green staining reveal proteoglycan and collagen deposition respectively. Scale bar: overview—1000 µm, center and edge—50 µm.

**Figure 6:**
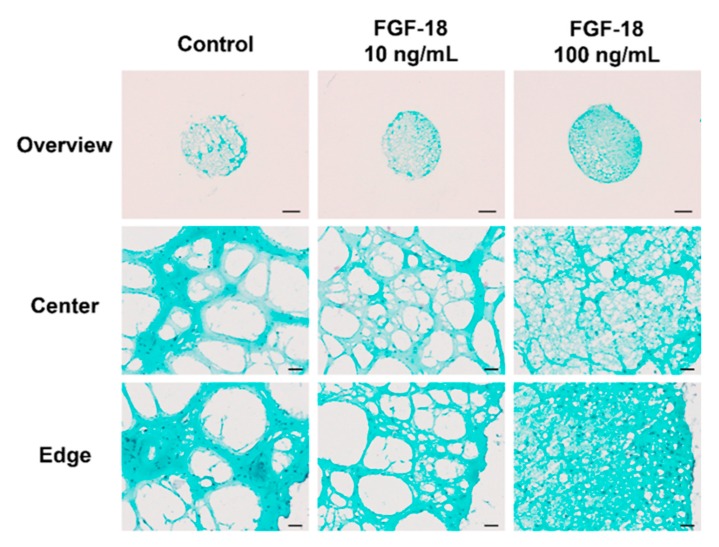
Representative Safranin O/Fast Green-stained transverse sections of human (mildly degenerated) nucleus pulposus cells encapsulated in FBG-HA hydrogel and stimulated with 0, 10, or 100 ng/mL FGF-18 after 14 days of culture. Scale bar: overview—1000 µm, center and edge—50 µm.

**Figure 7:**
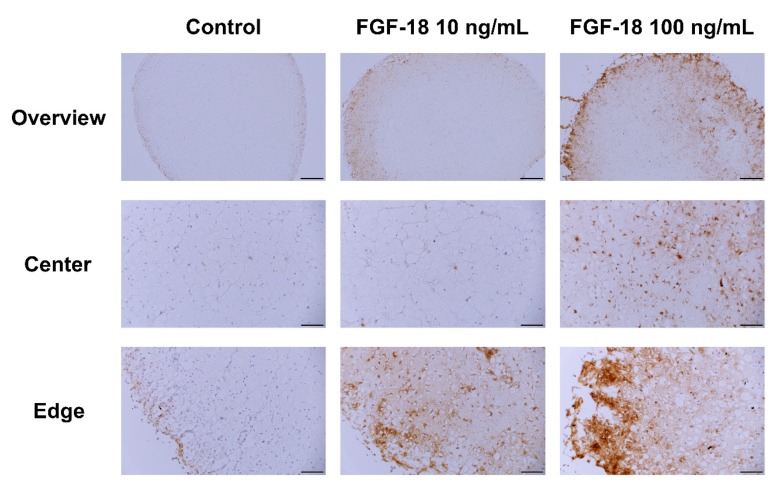
Aggrecan immunohistochemistry staining of bovine (healthy) nucleus pulposus cells encapsulated in FBG-HA hydrogel and stimulated with 0, 10, or 100 ng/mL FGF-18 after 14 days of culture. Scale bar: overview—500 µm, center and edge—100 µm.

**Figure 8:**
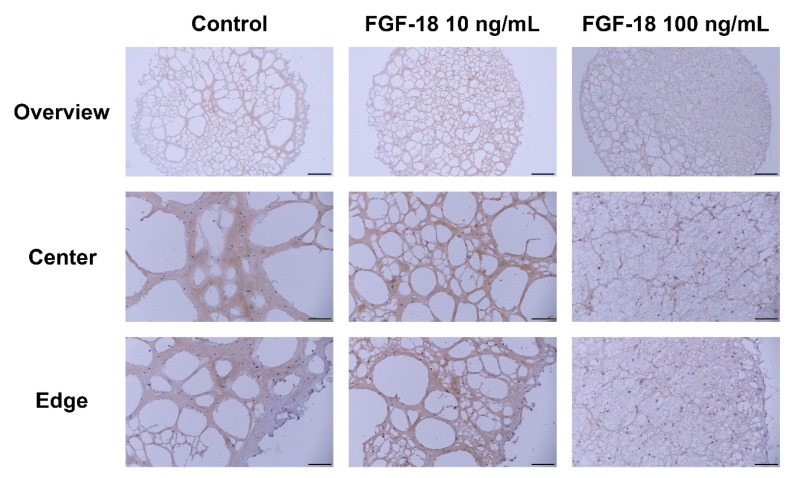
Collagen type II immunohistochemistry staining of human (mildly degenerated) nucleus pulposus cells encapsulated in FBG-HA hydrogel and stimulated with 0, 10, or 100 ng/mL FGF-18 after 14 days of culture. Scale bar: overview—500 µm, center and edge—100 µm.

Images of Safranin O/Fast Green-stained transverse sections of encapsulated NP cells in hydrogel for 14 days are shown in Figure 5 and Figure 6. Healthy bovine NP cells showed intense purple staining at the outer layer of the hydrogel, which indicated proteoglycan accumulation. An increased proteoglycan staining both in terms of staining intensity and size of the stained area was observed when incubated with 10 or 100 ng/mL FGF-18 (Figure 5). This was further confirmed by aggrecan immunohistochemistry staining in samples with bovine NP cells (Figure 7). Human mildly degenerated NP cells showed no difference in staining after 14 days in the FGF-18 treated group compared to the control. Consistent with the biochemical analysis, we did not detect any effect on proteoglycan staining by FGF-18 stimulation after 14 days, although hydrogels of the FGF-18 100 ng/mL group showed a slight increase in size and collagen staining intensity of the hydrogel (Figure 6). Collagen type II immunohistochemistry staining was performed in samples with human NP cells, which showed no difference in the staining intensity among the three groups (Figure 8).

## 3. Discussion

Various bioactive growth factors, as well as polymer-based hydrogels have been tested as non-invasive injectable treatments for IVD regeneration because of their capacity to support disc cell growth and metabolism. To our knowledge, this is the first study that has investigated the effect of FGF-18 on bovine and human NP cells.

FBG-HA hydrogel showed a translational potential as indicated by its positive modulatory effect on human mildly degenerated NP cells. FBG-HA conjugate-based hydrogels were found to support the survival and differentiation of bovine NP cells [33] and human chondrocytes [34]. In the current study, the FBG-HA hydrogel, which supplied a biomimetic ECM microenvironment, was used to encapsulate bovine and human NP cells for in vitro 3D culture studies. The hydrogel could maintain cell survival of human NP cells, as indicated by steady DNA content over 14 days of culture, whereas a decrease in DNA content was noticed in healthy bovine cell samples over time. Healthy bovine cells have a higher metabolic activity compared with degenerated human NP cells. Furthermore, it is known that fibrin degrades over prolonged cultures [35]. In contrast, FBG-HA hydrogel showed a potential regenerative effect on mildly degenerated human NP cells, as indicated by upregulation of COL2 gene expression and increased GAG release into the culture medium over 14 days of culture (Figure 3B and Figure 4F). The NP-specific cell phenotype markers CA12 [36] and KRT19 [37] showed a steady or slightly increased level in healthy bovine and mildly degenerated human NP cells, respectively, after 14 days culture in FBG-HA hydrogel (Figure 2A,B). This suggested that the specific cell environment given by the hydrogel could partially retain or regain the healthy NP phenotype, as it has been shown in former in vitro studies [24,33,38,39]. In cells derived from young and healthy animals, the NP GAG synthesis level was 10-fold higher compared with mildly degenerated human NP cells (Figure 4C–F). Upregulation of MMP and ADAMTS expression is implicated in IVD extracellular matrix degradation, leading to the development of IVD degeneration [40]. On the other hand, a high aggrecanase activity was observed in human NP tissue until the age of young juvenile, which may be related to the development or growth of tissue [41]. Bovine NP cells cultured in FBG-HA hydrogel did not show any significant change in MMP3, ADAMTS4, or ADAMTS5 gene expression after 14 days of culture. On the other hand, the human NP cells showed an upregulation of ADAMTS4 gene expression after 3D in vitro culture, which may indicate a degeneration or self-remodeling effect. Overall, these results indicated a difference in the behavior of bovine healthy NP cells and human mildly degenerated NP cells cultured in the same microenvironment. The present data indicate no difference between traumatic and degenerative human NP samples, which can be attributed to the equal degeneration grade. Nonetheless, because of our small sample size, we cannot make any definitive conclusions about differences between traumatic and degenerative NP.

In the performed in vitro studies, we could not observe a pronounced effect of FGF-18 for NP cells. Characterization of the FGF receptor expression on mRNA level showed that healthy bovine NP cells expressed FGFR3 receptors, which is congruent with former research work [42]. FGFR3 is the major signal transducer of FGF-18 [12,42]. We, therefore, tested a mutant of FGF-18 (FGF-18 ∆51) that has increased affinity to FGFR3 [23] for the stimulation of bovine NP cells. To our knowledge, no studies have been conducted so far on the expression of different FGFRs in bovine or human NP cells, seen in contrast to articular chondrocytes, which are relatively well-characterized for their FGFR expression [43]. FGFR3 expression in degenerated human NP cells was found to be highly variable and donor-dependent (independent from the grade of degeneration), whereby FGFR3 was not detected in 2 out of 4 donors after 7 or 14 days of culture. Therefore, FGF-18 wild type was used in the stimulation experiments on human NP cells. Dailey and colleagues stated that the activation of FGFR could induce different responses in different cell types, which might be caused by the ability of the receptors to elicit different signals (e.g., proliferation, differentiation, or apoptosis, etc.) by engaging the appropriate transducing molecule [44]. This might explain the regenerative effect of FGF-18 on human articular OA cartilage but not on human NP. The effect of the regulation of receptor expression in relation to their individual FGF-18 binding affinity needs further investigation.

FGF-18 did not show a significant effect on marker genes for regeneration, cell proliferation, or GAG synthesis in human or bovine NP cells. GAG accumulation after stimulation with 10 and 100 ng/mL FGF-18 in the periphery of bovine NP cell samples was observed after 14 days of culture, but not in human NP cell samples. This result could be due to the fact that the bovine NP cells were younger and metabolically more active compared with the mildly degenerated human NP cells [45]. Moreover, FGFR3 showed a higher gene expression in bovine NP cells compared to the degenerated human NP cells. The concentration of FGF-18 used in the current study (10 and 100 ng/mL) was comparable to other studies done with different FGFs on nucleus pulposus cells [42] and cartilage [46], while a direct conjugation to the FBG-HA hydrogel might enhance the potential regenerative effect of FGF-18 by controlled slow release. Further characterization of the FGFR protein expression on the cell surface would bring forth insightful information for the application of FGF-18 as a regenerative therapy. More pronounced matrix synthesis in mildly degenerated human NP cells might be observed in longer-term studies.

## 4. Materials and Methods

### 4.1. Materials and Reagents

An FBG-HA conjugate hydrogel (RegenoGel, available online: https://regenogel.co.il/, accessed on 24 October 2018) [33,47] was synthesized at ProCore Biomed Ltd. (Nes Ziona, IL). The conjugate was composed of FBG and HA in a 3.2:1 ratio, with a final concentration of 6.25 mg/mL FBG and 1.95 mg/mL HA. In comparison to hydrogels prepared from FBG alone or in a mixture of FBG and HA, FBG-HA conjugate hydrogels showed enhanced stability and mechanical properties, including reduced loss of water and shrinkage over time, as well as more pronounced viscoelastic properties [33,47].

FGF-18 mutant (FGF-18 ∆51) was provided by ProCore Bio-med Inc. (Nes Ziona, IL) and was used for stimulation of bovine NP cells, which were shown to express the FGFR3 gene. Because expression of FGFR3 could not be detected in all the donors of mildly degenerated human NP cells, the FGF-18 wild type was used for all experiments with human NP cells (FGF-18, human recombinant, cat. # 4082, BioVision, Inc. Milpitas, CA, USA). Both types of FGF-18 were reconstituted in phosphate-buffered saline (PBS) at a concentration of 10 µg/mL, and were further diluted with culture medium in the cell culture experiment. Heparin (Merck KGaA, Darmstadt, Germany) was added to the culture medium at a concentration of 1 µg/mL to activate the mitogenic activity of FGF-18 [12].

### 4.2. Methods

#### 4.2.1. Cell Isolation and In Vitro Culture in FBG-HA Hydrogel

Bovine NP cells were isolated from 8 to 12 months old caudal IVD, as described previously [33]. Human NP tissue was harvested, with ethical approval and the written consent of patients (ethical committee of the canton of Bern, Switzerland) with traumatically injured or degenerative IVDs, which were all classified as mildly degenerated (Pfirrmann grade 2–3, 30–55 years) [48]. Intervertebral disc degeneration is a natural process of aging, beginning as early as the age of 20 years old [49]. The traumatic samples collected in clinics are often mildly degenerated (i.e., Pfirrmann grade 2–3) and are comparable to degenerative IVD samples. Therefore, calf NP cells at the age of 8–12 months were used as healthy control. The collected human NP tissue was incubated with red blood cell lysis buffer for 5 min on a shaker and washed with PBS. The chopped tissue was then enzymatically digested with 0.2% w/v pronase in alpha minimum essential medium (αMEM) for 1 h, followed by 65 U/mL collagenase type II in αMEM/10% fetal bovine serum (FBS) for 12–14 h at 37 °C. The single-cell suspension obtained by filtering through a 100 µm cell strainer was seeded at a concentration of 10,000 cells/cm^2^ and expanded with αMEM/10% FBS under 2% O_2_. Expanded NP cells, bovine or human (passage 1–2), were encapsulated within FBG-HA hydrogel at a concentration of 6 × 10^5^ cells/gel for healthy bovine and 2 × 10^5^ cells/gel for mildly degenerated human NP cells. The gel was prepared by mixing 150 µL of FBG-HA conjugate solution containing NP cells with 10 µL of thrombin at room temperature, followed by a 30 min incubation at 37 °C to allow solidification. Cell–gel constructs were cultured in Dulbecco’s modified Eagle’s medium (DMEM, with 4.5 g/L glucose) with 2% FBS, 100 U/mL penicillin, 100 mg/mL streptomycin (1% Pen/Step) (all from Gibco, LifeTechnologies Limited, Paisley, UK), 50 mg/mL L-ascorbic acid 2-phosphate (Merck KGaA, Darmstadt, Germany), 1% ITS Premix (Discovery Labware, Aldrich, St. Louis, MO), and 1 µg/mL heparin (180 IU/mL, Merck KGaA, Darmstadt, Germany) for 7 or 14 days. The culture medium was changed every 2–3 days. FGF-18 ∆51 or rhFGF-18 was added to the culture medium at a concentration of 10 or 100 ng/ml.

#### 4.2.2. Biochemical Analysis

On day 7 and 14, cell–gel constructs were digested with 0.5 mg/mL proteinase K at 56 °C overnight. The GAG content in the hydrogels was measured using the 1,9-dimethylmethylene blue dye (DMMB) method [50] and was normalized to the DNA content in the respective samples. GAG content in hydrogels without cells on day 0 was subtracted from all samples. The DNA content in the hydrogels was measured with PicoGreen (Invitrogen, Eugene, OR, USA) according to the manufacturer’s instructions. The conditioned medium was collected at every medium change, and the GAG content was determined after 7 and 14 days in the pooled medium.

#### 4.2.3. Gene Expression Analysis

Total RNA was extracted from cell–gel constructs after 1, 7, and 14 days of culture. First, the gels were homogenized with TRI Reagent (Molecular Research Center MRC, cat. # TR-118, Cincinnati, OH) supplemented with 5 µL of Polyacryl Carrier (Molecular Research Center MRC, cat. # PC-152, Cincinnati, OH) in a tissue lyser (Qiagen, Hilden, Germany). The isolation was done by using a modification of the TRI Reagent procedure for the isolation of RNA from polysaccharide- and proteoglycan-rich sources. Reverse transcription was carried out with TaqMan Reverse Transcription Reagents (all from Applied Biosystems, Foster City, CA, USA) and 0.5 µg of total RNA. Real-time PCR was performed for collagen type I (COL1), collagen type II (COL2), aggrecan (ACAN), matrix metalloproteinase-3 (MMP3), a disintegrin and metalloproteinase with thrombospondin motifs 4 and 5 (ADAMTS4 and -5), carbonic anhydrase XII (CA12), keratin 19 (KRT19), and FGF receptors 1–4 (Table 1) using TaqMan Universal Master Mix (Applied Biosystems, cat. # 4324018, Carlsbad, CA). QuantStudio6 Flex Instrument and software (Applied Biosystems) were used for qPCR. Ribosomal protein lateral stalk subunit P0 (RPLP0) was used as the endogenous control. The comparative Ct method was performed for the relative quantification of target mRNA.

#### 4.2.4. Safranin O/Fast Green Staining and Immunohistochemistry

After 14 days of culture with or without FGF-18, FBG-HA hydrogel containing bovine or human NP cells were fixed in 70% methanol or 4% formalin, respectively, and transferred into PBS with 5% sucrose at 4 °C overnight before cryosectioning. Transverse cryosections were cut at a thickness of 8 µm through the central area of the hydrogel. Sections were stained with 0.1% Safranin O and 0.02% Fast Green to reveal proteoglycan and collagen deposition, respectively, and were then counterstained with Weigert’s Haematoxylin to reveal cell distribution.

Aggrecan immunohistochemistry staining was performed in samples with bovine NP cells [51]. After reduction and alkylation, sections were pre-treated with 0.025 U/mL chondroitinase ABC (Sigma-Aldrich Co., St. Louis, MO, USA) at 37 °C for 30 min, probed with aggrecan antibody (12/21/1-C-6, 5 µg/mL IgG, Developmental Studies Hybridoma Bank, University of Iowa) at 4 °C overnight, followed by incubation with a biotinylated horse anti-mouse secondary antibody, avidin-biotin-peroxidase (Vectastain Elite ABC kit, Vector Laboratories, city, state abbreviation if available, Burlingame, CA, USA), and ImmPACT® DAB Peroxidase (HRP) Substrate (Vector Laboratories). Collagen type II immunohistochemistry staining was performed in samples with human NP cells. Sections were pre-treated with 25 mg/mL hyaluronidase (Sigma-Aldrich Co.) and 0.25 U/mL chondroitinase ABC at 37 °C for 30 min, probed with collagen type II antibody (CIICI, 1 µg/mL IgG, Developmental Studies Hybridoma Bank, University of Iowa) at 4 °C overnight, followed by incubation with a biotinylated horse anti-mouse secondary antibody, avidin-biotin-peroxidase, and DAB. Negative control sections were incubated without the primary antibody. Sections were imaged in transmitted light using a Bx63 microscope (Olympus, Tokyo, Japan).

#### 4.2.5. Statistical Analysis

Graph Pad Prism V8 software (GraphPad Software, San Diego, CA, USA) was used for statistical analysis. One sample Kolmogorov–Smirnov test was used to define whether the data were normally distributed (normal distribution at *p* > 0.1). For data that were normally distributed, one-way ANOVA with Tukey’s post hoc test was used to determine differences among three or more groups, and unpaired *t*-test was used to determine differences between two groups. For data that were not normally distributed, Kruskal–Wallis test was used to determine differences among three or more groups, and Mann–Whitney U-test was used to determine differences between two groups. A *p*-value < 0.05 was considered statistically significant, and a *p*-value < 0.1 was considered to indicate a trend. Two technical replicates were performed in biochemical analyses, and three technical replicates were done for qPCR. Four different donors were used for bovine and human NP cells.

## 5. Conclusions

In conclusion, our results showed that the FBG-HA hydrogel supported the survival and positively modulated the phenotype of mildly degenerated human NP-derived cells within a biocompatible biomimetic 3D environment. The FBG-HA hydrogel might be a promising treatment option for NP regeneration and could serve as a carrier for a variety of growth factors and cells. Further preclinical studies are required to determine their therapeutic potential. By adding FGF-18, we could not see a significant effect on bovine and human NP cells at the tested experimental conditions.

## Figures and Tables

**Table 1:** Oligonucleotide primers and probes (bovine and human) used for qRT-PCR. Primers and probes with the sequence shown were custom-designed; primers and probes with the catalog number were from Applied Biosystems. fw: Forward, rev: Reverse, FAM: Carboxyfluorescein, TAMRA: Tetramethylrhodamine.

Gene	Primer/Probe Type	Sequence
bCOL1	Primer fw (5′–3′)	TGC AGT AAC TTC GTG CCT AGC A
	Primer rev (5′–3′)	CGC GTG GTC CTC TAT CTC CA
	Probe (5′FAM/3′TAMRA)	CAT GCC AAT CCT TAC AAG AGG CAA CTG C
bCOL2	Primer fw (5′–3′)	AAG AAA CAC ATC TGG TTT GGA GAA A
	Primer rev (5′–3′)	TGG GAG CCA GGT TGT CAT C
	Probe (5′FAM/3′TAMRA)	CAA CGG TGG CTT CCA CTT CAG CTA TGG
bACAN	Primer fw (5′–3′)	CCA ACG AAA CCT ATG ACG TGT ACT
	Primer rev (5′–3′)	GCA CTC GTT GGC TGC CTC
	Probe (5′FAM/3′TAMRA)	ATG TTG CAT AGA AGA CCT CGC CCT CCA T
bMMP3	Primer fw (5′–3′)	GGC TGC AAG GGA CAA GGA A
	Primer rev (5′–3′)	CAA ACT GTT TCG TAT CCT TTG CAA
	Probe (5′FAM/3′TAMRA)	CAC CAT GGA GCT TGT TCA GCA ATA TCT AGA AAA C
bADAMTS4	Primer fw (5′–3′)	CCC CAT GTG CAA CGT CAA G
	Primer rev (5′–3′)	AGT CTC CAC AAA TCT GCT CAG TGA
	Probe (5′FAM/3′TAMRA)	AGC CCC CGA AGG GCT AAG CGC
bADAMTS5	Primer fw (5′–3′)	GAT GGT CAC GGT AAC TGT TTG CT
	Primer rev (5′–3′)	GCC GGG ACA CAC CGA GTA C
	Probe (5′FAM/3′TAMRA)	AGG CCA GAC CTA CGA TGC CAG CC
bCA12	Primer fw (5′–3′)	CTG CCA GTC CGC AAT TTG T
	Primer rev (5′–3′)	CCC CGG ACC TGC ATG TC
	Probe (5′FAM/3′TAMRA)	CCA CTC AGT GAA GGT GAA ACT GCC CA
bKRT19		Bt03219428_m1
bFGFR1		Bt03258913_m1
bFGFR2		Bt03649230_m1
bFGFR3		Bt03259318_m1
bFGFR4		Bt04292959_m1
bRPLP0		Bt03218086_m1
hCOL1	Primer fw (5′–3′)	CCC TGG AAA GAA TGG AGA TGA T
	Primer rev (5′–3′)	ACT GAA ACC TCT GTG TCC CTT CA
	Probe (5′FAM/3′TAMRA)	CGG GCA ATC CTC GAG CAC CCT
hCOL2	Primer fw (5′–3′)	GGC AAT AGC AGG TTC ACG TAC A
	Primer rev (5′–3′)	GAT AAC AGT CTT GCC CCA CTT ACC
	Probe (5′FAM/3′TAMRA)	CCT GAA GGA TGG CTG CAC GAA ACA TAC
hACAN	Primer fw (5′–3′)	AGT CCT CAA GCC TCC TGT ACT CA
	Primer rev (5′–3′)	CGG GAA GTG GCG GTA ACA
	Probe (5′FAM/3′TAMRA)	CCG GAA TGG AAA CGT GAA TCA GAA TCA ACT
hMMP3		Hs00968305_m1
hADAMTS4		Hs00192708_m1
hADAMTS5		Hs01095518_m1
hKRT19		Hs00761767_s1
hFGFR1		Hs00241111_m1
hFGFR2		Hs01552918_m1
hFGFR3		Hs00179829_m1
hFGFR4		Hs01106910_g1
hRPLP0	Primer fw (5′–3′)	TGG GCA AGA ACA CCA TGA TG
	Primer rev (5′–3′)	CGG ATA TGA GGC AGC AGT TTC
	Probe (5′FAM/3′TAMRA)	AGG GCA CCT GGA AAA CAA CCC AGC
